# The Transfer of Sphingomyelinase Contributes to Drug Resistance in Multiple Myeloma

**DOI:** 10.3390/cancers11121823

**Published:** 2019-11-20

**Authors:** Sylvia Faict, Inge Oudaert, Ludovic D’Auria, Jonas Dehairs, Ken Maes, Philip Vlummens, Kim De Veirman, Elke De Bruyne, Karel Fostier, Isabelle Vande Broek, Rik Schots, Karin Vanderkerken, Johannes V. Swinnen, Eline Menu

**Affiliations:** 1 Department of Hematology and Immunology, Myeloma Center Brussels, Vrije Universiteit Brussel, UZ Brussel, B-1090 Brussels, Belgium; Sylvia.Faict@vub.be (S.F.); Inge.Oudaert@vub.be (I.O.); Ken.Maes@vub.be (K.M.); Philip.Vlummens@vub.be (P.V.); Kim.De.Veirman@vub.be (K.D.V.); Elke.De.Bruyne@vub.be (E.D.B.); Rik.Schots@uzbrussel.be (R.S.); Karin.Vanderkerken@vub.be (K.V.); 2 Neurochemistry Unit, Institute of Neuroscience, Université Catholique de Louvain, B-1200 Brussels, Belgium; ludovic.dauria@uclouvain.be; 3 Laboratory of Lipid Metabolism and Cancer, Department of Oncology, LKI-Leuven Cancer Institute, KU Leuven, B-3000 Leuven, Belgium; jonas.dehairs@kuleuven.be (J.D.); j.swinnen@kuleuven.be (J.V.S.); 4 Department of Clinical Hematology, Universitair Ziekenhuis Gent, B-9000 Ghent, Belgium; 5 Department of Clinical Hematology, Onze-Lieve-Vrouwziekenhuis Aalst, B-9300 Aalst, Belgium; Karel.Fostier@uzbrussel.be; 6 Department of Clinical Hematology, Algemeen Ziekenhuis Nikolaas, B-9100 Sint-Niklaas, Belgium; Isabelle.VandeBroek@aznikolaas.be

**Keywords:** lipidomics, drug resistance, exosomes, extracellular vesicles, multiple myeloma

## Abstract

Multiple myeloma (MM) is well-known for the development of drug resistance, leading to relapse. Therefore, finding novel treatment strategies remains necessary. By performing a lipidomics assay on MM patient plasma, we aimed to identify new targets. We observed a dysregulation in the sphingolipid metabolism, with the upregulation of several ceramides and downregulation of sphingomyelin. This imbalance suggests an increase in sphingomyelinase, the enzyme responsible for hydrolyzing sphingomyelin into ceramide. We confirmed the upregulation of acid sphingomyelinase (ASM) in primary MM cells. Furthermore, we observed an increase in ASM expression in MM cell lines treated with melphalan or bortezomib, as well as in their exosomes. Exosomes high in ASM content were able to transfer the drug-resistant phenotype to chemosensitive cells, hereby suggesting a tumor-protective role for ASM. Finally, inhibition of ASM by amitriptyline improved drug sensitivity in MM cell lines and primary MM cells. In summary, this study is the first to analyze differences in plasma lipid composition of MM patients and match the observed differences to an upregulation of ASM. Moreover, we demonstrate that amitriptyline is able to inhibit ASM and increase sensitivity to anti-myeloma drugs. This study, therefore, provides a rational to include ASM-targeting-drugs in combination strategies in myeloma patients.

## 1. Introduction

Multiple myeloma (MM) is a plasma cell malignancy in which the tumoral cells reside in the bone marrow. As such, it is one of the most common hematological malignancies, second only to non-Hodgkin lymphomas. MM is always preceded by a precursor state called monoclonal gammopathy of undetermined significance (MGUS) [1]. MM is notorious for its drug resistance, due to both extrinsic mechanisms through interaction with the bone marrow microenvironment, as well as intrinsic adaptations of the myeloma cells themselves [2]. So far, most efforts towards the identification of novel targets have focused on the molecular pathways on genomic and transcriptomic levels. 

In recent years, the lipid metabolism has become a more closely studied topic in the field of cancer [3]. Besides being the building blocks of cell membranes or energetic fuel, lipids are also known to serve as signaling molecules and are an integral part of cell metabolism. 

In this paper, we investigate the lipidome in MM patients with a focus on sphingolipids. 

These sphingolipids are of interest in cancer metabolism because of their importance in cell signaling transduction, ultimately leading to proliferation or apoptosis. Bioactive sphingolipids are, e.g., ceramides, sphingosine, and sphingosine-1-phosphate. Depending on the length of their fatty acid chain, their function changes greatly from stimulating proliferation to inducing apoptosis in cancer cells [4]. In breast cancer, Cai et al. found an increase in various ceramides (C16:0, C24:0, C24:1), translating to increased tumor progression and drug resistance [5]. 

Ceramide levels can increase by either catabolizing membrane-bound sphingomyelin by neutral or acid sphingomyelinase (SMase, a form of phospholipase C), or by de novo synthesis through ceramide synthase, starting from the condensation of serine and palmitoyl-CoA [6]. 

While neutral SMase is only active on the inner leaflet of the cell membrane, acid SMase (ASM) metabolizes sphingomyelin into ceramide on the outer leaflet of the membrane, as well as inside the lysosomal compartment [6]. 

Ceramide may act by the alteration of membrane biophysics, the self-association of ceramide molecules within the cell membrane and ultimately the formation of larger ceramide-enriched membrane domains/platforms [7]. As Carpinteiro et al. showed in a melanoma model, a higher secretion of ASM could result in the formation of ceramide-enriched membrane platforms on tumor cells, allowing integrins to cluster on these platforms and thus allowing diffuse metastasis of melanoma cells [8]. In multiple sclerosis, a higher secretion of ASM was attributed to the secretion of ASM-enriched exosomes in the cerebrospinal fluid of these patients [9]. Exosomes are a type of small extracellular vesicles (sEVs) formed inside multivesicular endosomes. They are actively secreted by cells via either the endosomal sorting complex required for the transport (ESCRT)-dependent pathway or the ESCRT-independent pathway, relying on ceramide generation by neutral sphingomyelinase 2 [10]. Our group has demonstrated that myeloma cells utilize exosomes for intercellular communication with their microenvironment, hereby actively increasing immune suppression [11], angiogenesis [12], osteolysis [13], and drug resistance [14]. This ultimately contributes to myeloma progression and the development of therapy-refractory disease.

In this paper, the lipidome of peripheral blood plasma of MM patients was investigated. We found that the lipidome of peripheral plasma in myeloma patients differed from healthy plasma: levels of several ceramide species were increased while levels of sphingomyelin were decreased. In vitro, we observed that the responsible enzyme ASM was expressed in several MM cell lines. Moreover, this ASM was actively secreted in MM exosomes, and conferred drug resistance to recipient cells. Resistance to standard-of-care agents bortezomib and melphalan could be abrogated by inhibiting ASM using amitriptyline, a tricyclic antidepressant commonly used to treat neuropathic pain in MM patients. Taken together, our data support the rationale of combining amitriptyline with front-line therapy in MM patients.

## 2. Results

### 2.1. MM Patients Show a Dysregulation in Lipid Metabolism with an Increased Amount of Ceramides and a Decrease in Sphingomyelin

**Figure 1:**
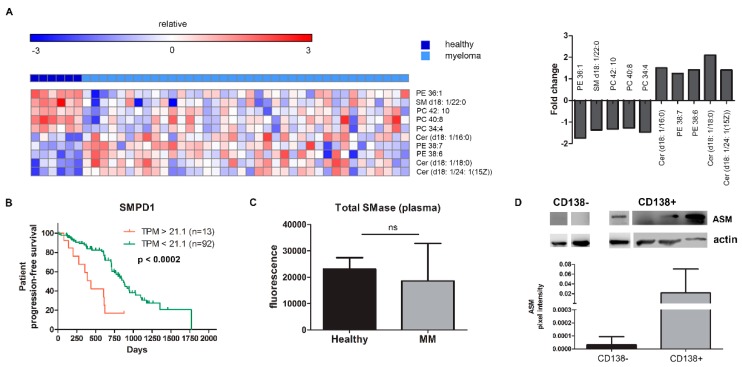
MM patients contain increased levels of ceramides and decreased levels of sphingomyelin. (**A**) Lipidomics analysis showing differences in plasma samples of healthy volunteers (n = 6) and multiple myeloma patients (n = 38) such as an increase of Cer16:0, Cer18:0, and Cer 24:1(15Z) and a decrease of SM 22:0. FDR adjusted *p* < 0.05 for fold changes. (**B**) Kaplan–Meier curve for progression-free survival (PFS) after MaxStat analysis of SMPD1 expression in MM patients (MMSET subgroup). (**C**) Total SMase levels measured in plasma samples of healthy volunteers (n = 6) and MM patients (n = 57). (**D**) Western blot of CD138 negative and positive fractions for the presence of acid sphingomyelinase (ASM). Four representative samples of CD138+ samples for a total n = 8. Immunoblot can be found in Supplementary Figure S4A. PE = phosphatidylethanolamine, SM = sphingomyelin, PC = phosphatidylcholine, Cer = ceramide, TPM = transcripts per million, SMase = sphingomyelinase, MM = multiple myeloma, ASM = acid sphingomyelinase.

A lipidomic assay was performed on plasma samples of MM patients (n = 38) and healthy controls (n = 6), revealing the dysregulation of several lipids, of which the top 5 up- and downregulated lipids, are shown as a heatmap in Figure 1A. The actual lipid concentrations are shown in Supplementary Figure S1A, demonstrating a similar trend. Furthermore, we also analyzed MGUS plasma samples (n = 4), and by principal component analysis found that the lipid profile of MGUS patients is intermediate between the healthy group and myeloma group (Supplemantary Figure S1B). 

Comparing healthy samples vs. MM samples, we observed a significant upregulation of phosphatidylethanolamine (PE) in two species (38:7; 38:6) and a downregulation in another (PE 36:1). More importantly, three ceramide species (d18:1/16:0), (d18:1/18:0), and (d18:1/24:1(15Z)) were upregulated with a 1.5 to 2-fold increase in MM, while sphingomyelin (SM (d18:1/22:0)), the sixth most frequent sphingomyelin species, was significantly downregulated (Figure 1A and Supplementary Figure S1A). Patient and disease characteristics are provided in the supplementary materials, Table S1. We observed no difference in lipid composition between newly diagnosed MM and relapsed/refractory MM samples.

In view of the well-known role of the enzyme sphingomyelinase (SMase) in the conversion of sphingomyelin into ceramide [6], we postulated that an upregulation of this enzyme in MM patients could lead to the imbalance in ceramides and sphingomyelins. 

We first determined the clinical impact of the different SMases by analyzing the correlation of gene expression levels of both neutral (SMPD2-4) and acid SMase (SMPD1) with progression-free survival (PFS) using the CoMMpass IA12 dataset released by the MMRF. In the subgroup of patients overexpressing the MMSET gene, we see a negative impact of the presence of SMPD1, resulting in an ultra-high-risk profile of patients overexpressing both MMSET and SMPD1 (Figure 1B). SMPD2 and SMPD4 overexpression also result in a worse PFS in this myeloma subgroup (Supplementary Figure S1C).

We next quantified the amount of total SMase in the peripheral plasma of myeloma patient samples. Compared to healthy controls, we did not see an increase in the peripheral plasma of MM patients (Figure 1C). Moreover, a waterfall plot of the total SMase of individual samples, based on ISS stages, did not discern any differences (Supplementary Figure S1D). However, SMase might only be increased in the tumor cells themselves. We indeed found that both total and acid SMase (ASM) were present in the CD138+ MM cells isolated from the bone marrow in 63% of patients (n = 8). In contrast, in the CD138- fraction, representing the non-clonal, non-malignant cell fraction of the bone marrow, this enzyme was only marginally detected (n = 3; Figure 1D).

**Figure 2:**
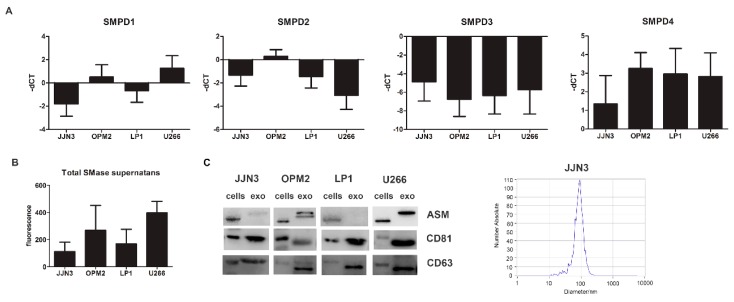
SMPD1 expression levels correlate with total SMase content in supernatant in MM cell lines and their exosomes. (**A**) Basal-dCT values of all four genes coding for SMases (SMPD1–4) measured by qRT-PCR in four human MM cell lines (JJN3, OPM2, LP1, and U266) after 24 h of culture. The housekeeping gene ABL1 was used for data normalization, and differential gene expression was determined using the comparative ΔΔCt method. (**B**) Basal total SMase content determined in supernatans by an AmplexRed SMase assay after 24 h of culture for all four cell lines. The amount of SMase activity measured in these cell lines corresponded best to SMPD1 expression, coding for acid SMase (ASM). (**C**) Cells and exosomes were isolated after 24 h of culture and analyzed for ASM content and exosomal markers, CD81 and CD63. Nanoparticle tracking analysis by Zetaview analysis shows a mean diameter of 50–150 nm, confirming the expected size of exosomes. Immunoblot can be found in Supplementary Figure S4B. ASM = acid sphingomyelinase, exo = exosomes. In A and B, the averages of n > 3 are shown.

Next, we determined the presence of neutral and acid sphingomyelinase mRNA as well as total secreted and cellular SMase in human multiple myeloma cell lines (HMCL) representing different genetic subtypes of MM, including JJN3 (c-Maf), LP1 (MMSET/FGFR3), OPM2 (MMSET/FGFR3), and U266 (CCND1). SMPD1 and SMPD4 were among the most expressed genes, respectively, coding for ASM and neutral SMase 3 (Figure 2A). The secreted SMase amount measured in the supernatant after 24 h of cell culture (Figure 2B) was most consistent with the mRNA levels of SMPD1, coding for ASM. Therefore we focused further on ASM and determined whether ASM could also be packaged into sEVs, or exosomes, similar to what was described for exosomes in the cerebrospinal fluid of multiple sclerosis patients [9]. The ASM content in the exosome enriched fraction differed from cell line to cell line. Both OPM2 and U266 had remarkable higher quantities of ASM inside their vesicles than both JJN3 and LP1. The isolated sEVs were less than 150 nm and were positive for tetraspanins CD63 and CD81 (exosome markers), and as such can be considered exosomes (Figure 2C and Supplementary Figure S2A). 

### 2.2. Standard-of-Care Drugs Melphalan and Bortezomib Induce a Higher Expression in ASM in MM Cells and Their Exosomes 

Melphalan and bortezomib are used very effectively in the first-line treatment of MM patients. Unfortunately, many of the patients who first responded well to treatment will face a disease relapse whereby the tumor cells are no longer sensitive to these drugs. 

**Figure 3:**
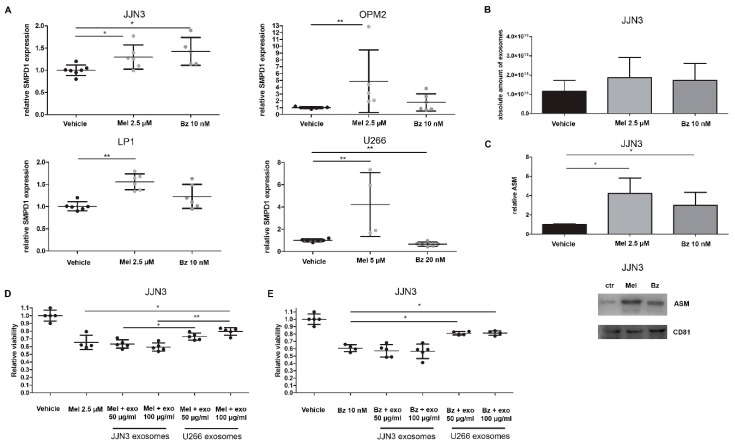
Standard-of-care drugs melphalan and bortezomib increase acid SMase expression and protein levels in MM cell lines and their exosomes, which contributes to drug resistance development. (**A**) SMPD1 levels were measured by qRT-PCR in MM cell lines JJN3, OPM2, LP1, and U266, which were treated for 24 h with melphalan (Mel) and bortezomib (Bz). (**B**) Nanoparticle tracking analysis shows the amount of exosomes secreted after 24 h treatment with Bz and Mel. (**C**) Exosomes isolated from Mel or Bz treated JJN3 cells were analyzed and quantified by Western blot for ASM and exosomal marker CD81. Immunoblot can be found in Supplementary Figure S4C. (**D,E**) JJN3 cells were treated with either 2.5 µM melphalan (**D**) or 10 nM bortezomib (**E**) with or without previously isolated JJN3 (ASM-low) or U266 (ASM-high) exosomes. After 24 h of culture, cell viability was measured by a CellTiter Glo. Mel = melphalan, Bz = bortezomib, ASM = acid sphingomyelinase, exo = exosomes. *p* < 0.05 (*), and *p* < 0.01 (**) were considered statistically significant. The averages of n > 3 are shown.

First, we determined whether ASM expression was increased in response to drug treatment. We focused on the effects of melphalan (Mel) and bortezomib (Bz) treatment on ASM expression, both in HMCL and their exosomes. HMCL were treated with low doses of melphalan and bortezomib in medium without serum. After 24 h, the expression level of SMPD1 was measured by qRT-PCR (Figure 3A). OPM2 and U266 cells increased their ASM expression in response to melphalan treatment four-fold, while JJN3 and LP1 cells had a smaller but still significant response in ASM expression to melphalan. Treatment with bortezomib induced a significantly higher ASM expression in the JJN3 cells while there was a slight increase in LP1 and OPM2 cells. 

When comparing the different myeloma cell lines, we observed that the U266 cells are most resistant to therapy, as the IC50 of both melphalan and bortezomib is higher (Supplementary Figure S2B). This seems to correlate with high ASM mRNA expression and ASM protein levels in supernatant and exosomes (Figure 2A–C). Moreover, bortezomib treatment did not induce any increase in SMPD1 expression.

Next, we investigated the effects of these standard-of-care (SoC) drugs on exosome secretion and content. We treated 450 million JJN3 cells with sub-lethal doses of melphalan and bortezomib, and isolated their sEVs after 24 h of treatment. By nanoparticle tracking analysis (NTA), we observed a small, non-significant increase in the number of small particles (Figure 3B), while the mean diameter of these particles remained roughly the same (Control: 85 nm, Mel: 98 nm, Bz: 90 nm). Next, we analyzed the sEVs secreted from the same number of cells by Western blot analysis. We again confirmed the presence of the exosomal tetraspanin CD81 (Figure 3C). Moreover, we saw an upregulation of CD81 in Mel and Bz-treated cells, mirroring the results obtained by NTA and confirming the fact that Mel and Bz induce a higher exosome secretion by JJN3 cells. Interestingly, after normalization to the exosome number, the ASM content of isolated sEVs was also increased in Mel and Bz treated cells (Figure 3C). 

As it seems that drug resistance in U266 cells is partially mediated by high ASM levels, we next evaluated whether this resistance could be transferred to receptor cells. Therefore, we transferred exosomes/sEVs from U266 cells (which are less sensitive to Mel and Bz) to the Mel and Bz sensitive JJN3 cells. As a control, we used autologous JJN3 exosomes, which contain about 100-fold less ASM content than U266 exosomes. As shown in Figure 3D,E, U266 sEVs, which are high in ASM content, provide protection to both Mel and Bz treatment in JJN3 cells and induce a relative increase of viability of about 25%, whereas JJN3 sEVs do not. This suggests that U266 exosomes can transfer the drug resistance of their parental cells to other MM cells by supplementing them with ASM. Moreover, when either JJN3 cells themselves or U266 cells were treated with amitriptyline before exosome isolation, this effect was countered (Supplementary Figure S2C).

### 2.3. Inhibiting SMase in MM Cells Strengthens the Effects of MM Treatment by Upregulating the PARP and Caspase 3 Pathways

Since drug resistance seems to be correlated with a higher expression of SMase, and this resistance can be provoked by the transfer of exosomes, we next tried to inhibit SMase to increase drug efficacy. 

For the inhibition of ASM, we chose the drug amitriptyline, which is a tricyclic antidepressant often used in MM patients to treat side effects of neuropathy induced by either bortezomib or thalidomide treatment. For the inhibition of neutral SMase, we used GW4869. In our previous work, we have already shown the efficacy of this drug in combination with bortezomib in vivo in a murine model by inhibiting exosome secretion via the ceramide-pathway [13]. 

**Figure 4:**
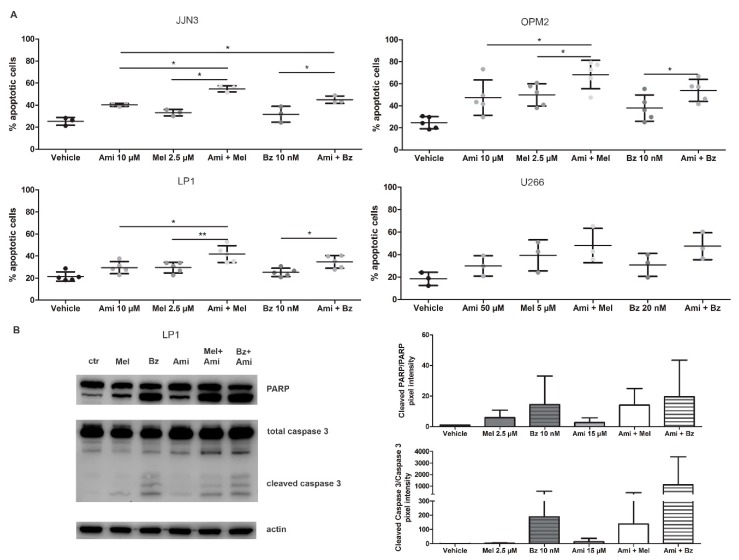
Amitriptyline, an acid SMase inhibitor, increases the drug efficacy of melphalan and bortezomib in MM cell lines by inhibiting ASM and inducing apoptotic cell death. (**A**) JJN3, OPM2, LP1, and U266 were treated with melphalan, bortezomib, and/or amitriptyline. After 24 h of culture, apoptotic cell levels were measured by flow cytometry with staining for 7-AAD and annexin V-FITC. (**B**) LP1 cells were treated with melphalan, bortezomib, and amitriptyline. After 24 h, cell lysates were isolated, and (cleaved) PARP and caspase 3 levels were verified and quantified by Western blot analysis. Non-significant effects were observed. Immunoblot can be found in Supplementary Figure S4D. Ctrl = control, Ami = amitriptyline, Mel = melphalan, Bz = bortezomib. *p* < 0.05 (*), and *p* < 0.01 (**) were considered statistically significant. The averages of n > 3 are shown.

HMCL (JJN3, LP1, OPM2, U266) were treated with Mel or Bz in combination with amitriptyline or GW4869 for 24 h and checked for apoptosis by flow cytometry. For JJN3, LP1, and OPM2 cells, combination therapy of Mel or Bz with amitriptyline (Figure 4A and Supplementary Figure S2D) or GW4869 (Supplementary Figure S3A) induced more cell death when compared to the single agents, with a relative increase in apoptotic cells of 25% to 30%. Again, U266 cells only responded to high levels (50 µM) of amitriptyline, underscoring their resistant nature. To determine whether amitriptyline can work synergistically with either SoC drug, combination indexes were calculated for JJN3 and OPM2 cells (Supplementary Figure S3B). For the JJN3 cells, amitriptyline at the highest concentration (50 µM) had a synergistic effect (combination index, CI < 0.8) on melphalan and bortezomib treatment, although effects are more pronounced on the melphalan treatment. For the OPM2 cells, high dose-amitriptyline showed a rather additive effect on SoC treatment, with only one synergistic effect observed when Ami 50 µM was combined with Mel 1 µM.

To verify the downstream effects of amitriptyline on these cells, treated cells were analyzed by Western blot. As seen in Figure 4B, the combination therapy of Mel or Bz with amitriptyline shows an increase in both cleaved PARP and cleaved caspase 3. 

Importantly, when analyzing the secreted amount of lipids by these cells, we see an increase of sphingomyelin when treating cells with amitriptyline in all conditions. This confirms the effect of amitriptyline on the inhibition of SMase, resulting in higher sphingomyelin content (Supplementary Figure S3C) Also, we did not observe a change in exosome secretion after treatment with amitriptyline, indicating that the observed effects induced by amitriptyline are likely a direct effect and not due to differential exosome secretion (Supplementary Figure S3D). 

**Figure 5:**
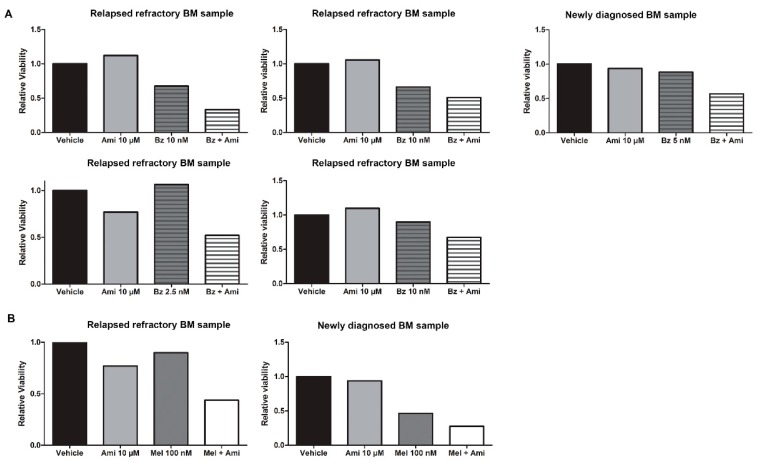
Amitriptyline stimulates drug efficacy of bortezomib and melphalan in primary MM bone marrow samples. (**A**) Primary MM cells were treated for 24 h with bortezomib/melphalan and amitriptyline, after which cell viability was assessed by CellTiter Glo (n = 5). (**B**) Primary MM cells were treated for 24 h with bortezomib/melphalan and amitriptyline, after which cell viability was assessed by CellTiter Glo (n = 2).

Finally, we confirmed the efficacy of the combination of amitriptyline and bortezomib or melphalan in primary cells obtained from the bone marrow of relapsed/refractory MM patients. We isolated the CD138+ fraction from bone marrow samples, treated them immediately with varying doses of Bz (n = 5) and Mel (n = 2), and added amitriptyline as a single agent (10 µM) or in combination with either Bz or Mel. When using low doses of either drug, we observed an increased drug effect of 24% to 50%, depending on the patient sample, by adding amitriptyline (Figure 5A,B).

## 3. Discussion

In this paper, we explored the lipid alterations in MM to unravel the role of these changes in drug resistance. We found an increase in the amount of ceramide in the plasma of MM patients, which correlated with an increased expression of acid sphingomyelinase (ASM) in MM cells. Importantly, ASM was upregulated by treating MM cells with standard-of-care drugs melphalan and bortezomib. Moreover, MM exosomes high in ASM content could induce resistance to these drugs, while inhibiting ASM resulted in an increased sensitivity. 

We performed a lipidomic analysis of patient plasma to determine which lipids are altered in MM. We found that ceramides are upregulated in patient plasma compared to age-matched healthy controls, at the expense of sphingomyelin. 

When analyzing an additional four MGUS patients, we could see in the PCA **(**Supplementary Figure S1B) of the lipid profile that these patients form an intermediate group between healthy and MM samples. This suggests that some, but not all, of the lipid alterations are already present in this premalignant stage; however, our sample size of MGUS patients is too small to draw exact conclusions on which lipids were already dysregulated and which were not. It bears no surprise that some of the lipid changes can already be observed in the MGUS stadium of MM since the same holds true for genetic changes. Our patient population contained newly diagnosed as well as relapsed refractory patients. There were no obvious differences in plasma lipid composition between both groups, and as such, it would seem that myeloma therapy does not influence lipid composition directly. 

A dysregulation in the lipid profile of plasma has been described for some forms of cancer; however, only very limited data is available for hematological malignancies. Our study is the first to uncover differences in the sphingolipid profile of MM patient plasma. In the future, it would be interesting to perform a lipidomic analysis on a larger number of MM patients, which would ideally also include MGUS and SMM patients to study the evolution at the level of lipid metabolism from MGUS to MM. 

To determine whether the altered lipids in the plasma truly originate from the MM cells, the lipid constitution of the MM cells should be compared to healthy counterparts. However, healthy plasma cells are difficult to isolate. We did compare for six patients the lipid profile of the peripheral plasma to CD138+ plasma cells isolated from the bone marrow [15]; however we found no correlation between these samples. This is not so surprising since the composition of plasma differs considerably from the composition of cells or tissue. Moreover, ceramide in the peripheral plasma is mainly associated with extracellular vesicles, and since MM is associated with higher levels of circulating EVs derived from the MM cells themselves, the alteration in the plasma lipid profile might be the result of a higher concentration of EVs in the plasma [16,17,18,19]. Other studies have also performed lipidomic analysis on patient plasma to discover potential biomarkers for solid tumors, such as lung, breast, pancreas, ovarian, and colorectal cancer. Especially, breast and lung cancer patients had significantly higher levels of sphingolipids and glycerophospholipids than healthy controls [20]. 

When examining the ceramide pathway, we hypothesized that the sphingomyelin/ceramide dysregulation is possibly due to a higher expression of SMase in MM patients. However, a change in SMase quantity in the MM cells might not necessarily be reflected in an increase in SMase in the peripheral plasma. Indeed, in 62.5% of CD138+ MM cells isolated from bone marrow samples, we could measure an increase in ASM compared to the other BM cells (CD138−), while the total SMase in plasma from healthy and MM patients did not vary significantly. When examining the expression of the different SMase subtypes in the overall MMRF patient cohort, we saw that especially for SMPD4, coding for neutral SMase 3 consistently entailed a worse prognosis (data not shown). However, when we focused on the aggressive genetic subtypes of MM, we found that in the MMSET subgroup, the presence of both SMPD1 and SMPD4 separately present a group of ultra-high-risk MM with a PFS of about one year. MMSET is the molecular target of the t(4;14)(p16;q32) translocation, which is one of the most common occurring translocations in MM, associated with a very poor prognosis [21,22]. 

Both SMPD1 (acid SMase, ASM) and SMPD4 (neutral SMase 3) were most expressed in four different MM cell lines. The profile of total secreted SMase was most consistent with the expression of SMPD1 or ASM. SMase generates ceramide by hydrolyzing sphingomyelin molecules. The resulting ceramide molecules can form ceramide-enriched membrane platforms, which will cluster specific receptors and enrich intracellular signaling molecules. This leads to an increase of the initiating signal, eventually generating a transmission of the signal into the cell. In this way, ceramide is involved in many cellular processes such as apoptosis, inflammation, and autophagy. [23].

ASM has been described in extent for its role in autophagy in cancer [24]. The enzyme can be activated by various stimuli, including infections, stress stimuli, and reactive oxygen species [23]. Both pro-apoptotic [25,26] as well as tumor protective roles have been attributed to this enzyme. 

The presence of ASM in MM cells is reflected in the amount of ASM present in the exosomes isolated from these MM cells. Exosomes are actively secreted by the cells, and often mirror the contents of their originating cell, both in lipid, protein, and nucleic acid content [27]. In MM, it was recently described that drugs such as bortezomib and melphalan induce a stimulation of exosome-secretion. These so-called chemoexosomes differ substantially from control exosomes in their protein content, with some proteins being up- or downregulated and others being exclusively present in either chemoexosomes or control exosomes. Bandari et al. described that these chemoexosomes are rich in heparanase-content, thereby altering the extracellular matrix and ultimately contributing to drug resistance [28].

The stimulation in exosome secretion as the result of intracellular stress could be a way for the cells to dispose of any waste material, or at the same time, communicate with neighboring cells [27]. In our results, we confirmed that exosome release is stimulated by anti-myeloma drugs, and particularly observed that the ASM content of the exosomes was increased. The increased presence of ASM in the MM cells and their exosomes upon treatment could reflect a tumor-protective effect of ASM by stabilizing cancer lysosomes and increasing autophagic flux [24,29].

To further investigate the effect of ASM-rich exosomes on myeloma cells, we added ASM-high exosomes from U266 cells unto ASM-low JJN3 cells, simultaneously treating the cells with bortezomib or melphalan. We noticed increased viability after adding the U266 exosomes, but not when adding the JJN3 exosomes. Therefore, U266 cells which are more resistant to both melphalan and bortezomib treatment than JJN3 cells seem to be able to transfer their drug resistance to these JJN3 cells through exosomes, which are high in ASM content. Moreover, treating U266 cells with amitriptyline prior to exosome collection, abrogated this effect. Furthermore, we also investigated the effects of LP1 exosomes on the bortezomib response in JJN3 cells since LP1 cells and exosomes express low levels of ASM but are also relatively resistant to bortezomib. These LP1 exosomes did not induce resistance to bortezomib in JJN3 cells [30]. 

Petersen et al. demonstrated that inhibition of ASM using cationic amphiphilic drugs (CAD, such as amitriptyline) led to lysosomal cell death in cancer cells, including ovarian, breast, prostate, cervix, and bone cancer cell lines [29]. These inhibitors of ASM were proven to be effective in a multidrug-resistant type of prostate cancer cells, and could even resensitize them to standard chemotherapy. They demonstrated that CAD accumulates inside the lysosome and inhibits ASM, leading to sphingomyelin accumulation in the lysosomes, which then leads to a leakage of lysosomal proteases into the cell by permeabilization of the lysosomal membrane. Interestingly, cancer cells are more sensitive to these drugs because their lysosomes are already less stable [29]. Amitriptyline was initially introduced by Merck in 1961 as a tricyclic antidepressant with strong antidepressant activity owing to its potent serotonin–norepinephrin reuptake inhibitor activities [23]. Several years later, its activity as an inhibitor of ASM was described [29,31,32]. In a more recent study, Gulbins et al. demonstrated that amitriptyline actually executes its antidepressant function by the ASM/ceramide pathway, leading to neuronal proliferation and survival rather than a direct effect on neurotransmitter reuptake [33]. Other functional inhibitors of ASM include other tricyclic antidepressants, antihistamines, and calcium channel blockers [29]. Here, we demonstrated that amitriptyline could increase the efficacy of the SoC drugs, bortezomib and melphalan. Others have also shown that amitriptyline induces an extended survival of myeloma-bearing mice [34]. Amitriptyline is a particularly attractive drug in MM treatment since it is often used to treat neuropathic pain elicited by anti-myeloma treatment with proteasome inhibitors (e.g., bortezomib) or immunomodulatory drugs (e.g., thalidomide). Because of its long-standing use, amitriptyline has a well-known side effect profile, is cheap, and easily accessible. 

Finally, we also inhibited neutral SMase 2 by using GW4869. Here too, we saw a more pronounced effect of SoC drug treatment. GW4869 is also used for blocking exosome secretion via the ceramide-pathway [27], therefore, we cannot exclude exosome-dependent effects. It is known that blocking the neutral sphingomyelinase pathway blocks exosome secretion in some, but not all, cell lines. However, these results suggest a role for both acid and neutral SMase in MM cells, both ultimately increasing ceramide production. From a translational point of view, amitriptyline is the more interesting choice of the two since it is already used on MM patients.

## 4. Materials and Methods 

### 4.1. Cell Lines 

The human MM cell lines (HMCL) JJN3, LP1, OPM2, U266 were obtained from ATCC (Molsheim, France). The identity of the cell lines was regularly checked by short-tandem repeat analysis. Cell lines were regularly tested for mycoplasma contamination and passaged no more than one month prior to experiments. Cells were cultured in RPMI-1640 medium (Lonza, Basel, Switzerland) supplemented with 10% fetal bovine serum (FBS) (Hycone, Logan, UT, USA), 2 mM L-glutamine and 1% penicillin/streptomycin (Thermo Fisher Scientific, Waltham, MA, USA) at 37 °C in 5% CO_2_. 

### 4.2. Isolation of CD138+ Cells from Bone Marrow Samples 

Mononuclear cells were isolated from freshly obtained bone marrow samples by using density gradient centrifugation with Lymphoprep™ (Catalog # 07801, STEMCELL™ technologies, Grenoble, France). CD138+ cells were selected by positive selection using human CD138 MicroBeads (Catalog # 130-051-301, Miltenyi Biotec, Gladbach, Germany) according to the manufacturer’s instructions. 

### 4.3. Lipidomic Analysis 

Bone marrow and blood samples were collected for routine diagnostic or evaluation purposes after patients’ written informed consent and in accordance with the Declaration of Helsinki and institutional Ethical board approval (B.U.N. 143201628501) from UZ Brussels University hospital.

Blood was sampled in EDTA tubes at the time of diagnosis or disease relapse at morning time, and platelet-free plasma was collected by double centrifugation at 1200× *g* for 10 min, after which supernatant was collected, snap-frozen, and stored air-tight at −80 °C. Samples were analyzed in one analytical batch, eliminating batch-to-batch variations. 

Targeted lipidomics were performed. Phospholipid species were analyzed by electrospray ionization tandem mass spectrometry (ESI-MS/MS) on a hybrid triple quadrupole/linear ion trap mass spectrometer (4000 QTRAP system; Applied Biosystems SCIEX) equipped with a TriVersa NanoMate (Advion Biosciences) robotic nanosource for automated sample injection and spraying. Briefly, lipids were extracted by mixing 700 μL of sample (100 μL of plasma diluted in PBS) with 800 μlL 1 N CH3OH:HCl 8:1 (*v/v*), 900 μL CHCl3, and 200 μg/mL of the antioxidant 2,6-di-tert-butyl-4-methylphenol. The organic phase was evaporated using a Savant Speedvac spd111v (Thermo Fisher Scientific), and the remaining lipid pellet was stored under argon at –20 °C. Mass spectrometry analysis of the extracted lipids was performed as described previously [35,36]. 

Differences in the lipid species in the plasma of healthy donors and MM patients were calculated using marker analysis in GENE-E software. We calculated the signal-to-noise statistic, *p*-value and Benjamini-Hochberg false discovery rate (FDR). The top 5 hits with FDR < 0.05 in either direction were selected and shown as a heatmap using GENE-E software. Principal component analysis (PCA) was performed on lipidomic profiles of healthy donors, MGUS, and MM patients. Normalized data were analyzed in R using the Clustvis package [37]. 

The lipidome datasets analyzed during the current study is available from the corresponding author on reasonable request.

### 4.4. CoMMpass Patient Sample Analysis 

The MMRF CoMMpass Trial (NCT01454297) is a longitudinal study of multiple myeloma patients in which genomic data is collected at diagnosis and relapse. The sequencing and clinical data, including survival information, are publically available through the MMRF research gateway portal (https://research.themmrf.org) [38]. We used the Interim Analysis 12 data which consists of 767 patient bone marrow samples taken at diagnosis for which both survival data and RNA sequencing data are available. We correlated gene expression levels with survival information to analyze the prognostic value of genes of interest using the MaxStat R package as previously described [39]. 

### 4.5. Drugs and Reagents 

Bortezomib and amitriptyline were purchased from Selleckchem (Munich, Germany), GW4869 and melphalan were purchased from Sigma-Aldrich (St-Louis, MO, USA). Bortezomib and amitriptyline were dissolved in dimethylsulfoxide while melphalan was dissolved in ethanol with a few drops of HCl, all according to the manufacturer’s instructions. GW4869 was also dissolved in DMSO, resuspended, and vortexed, after which the solution was incubated at 37 °C for 15 min to reach maximum solubility. 

### 4.6. Isolation and Characterization of Small EVs

JJN3, LP1, OPM2, and U266 cells were cultured without serum for 24 h and conditioned medium was collected after centrifugation and filtered using a 0.22 µM pore filter. The filtered medium was concentrated using a 150 kD Protein Concentrator (Thermo Scientific, Waltham, MA, USA) and filtered again with a 0.22 µM pore filter. 

From this concentrated conditioned medium, small EVs were isolated using a Exoquick-TC exosome precipitation solution (System biosciences, Mountain View, CA, USA) according to the manufacturer’s instructions, with the addition of a final high speed centrifugation step (10 000× *g*, 2 min) to eliminate contaminating cell debris. The concentration of EV proteins was determined by BCA protein analysis (Thermo Scientific). The size and number of EVs was determined by nanoparticle tracking analysis using Zetaview® NTA (Particle-Metrix, Germany), with the average size and number calculated from 11 independent replicates using NTA. Western blot analysis was used to confirm the presence of exosomal tetraspanins, CD63 and CD81. 

### 4.7. Viability and Apoptosis Assay 

OPM2, U266, JJN3, and LP1 cell lines were seeded at 500,000 cells/ml in RPMI-1640 medium without serum, supplemented with 2 mM L-glutamine and 1% penicillin/streptomycin. Cells were treated with 2.5 µM (for OPM2, JJN3, and LP1) or 5 µM (U266) melphalan, 10 nM (OPM2, JJN3, and LP1) or 20 nM (U266) bortezomib, 10 µM (OPM2, JJN3) or 15 µM (LP1, U266) GW4869 and 10 µM amitriptyline (OPM2, U266, JJN3, and LP1). Viability was measured after 24 h by a CellTiter Glo Luminescent Cell Viability Assay (Promega, Madison, WI, USA). Luminescence was measured using a Glomax luminometer (Promega). Viable cells were quantified after 24 h by AnnexinV-FITC staining (BD Biosciences, Belgium) and 7-AAD staining (BD Biosciences) by Flow Cytometry analysis on a FACSCanto flow cytometer (BD Biosciences). 

### 4.8. Quantitative Real-Time PCR 

RNA was isolated using the RNeasy kit (Qiagen) and 1 µg of RNA was converted to cDNA by the Verso cDNA Synthesis Kit (ThermoFisher Scientific, Waltham, MA, USA). Gene-specific primer sequences were as follows: SMPD1: forward (5’ – TGC CAG GTT ACA TCG CAT AG – 3’), reverse (5’ – AGG TTG ATG GCG GTG AAT AG – 3’); SMPD2: forward (5’ – CTT ACC CAG CAC ATC TAC ACT C – 3’), reverse (5’ – GAG CAC CAT GCC ACT TAG AT – 3’); SMPD3: forward (5’ – ATA CCC ACC ACC TAC GAG AA – 3’), reverse (5’ – GAA AGC CGA GAA ACG CAA AG – 3’), SMPD4: forward (5’ – CCC ACA GTG GTT TGC TAA GA – 3’), reverse (5’ – TTT CAG GCT AGC CAG TAG AAA G – 3’).

Real-time PCR was performed using SYBR Green (PowerUpTM SYBRTM Green Master Mix, Applied Biosystems) in a final volume of 25 µL, consisting of 1 µL of cDNA, 12.5 µL of SYBR Green, 1 µL of Primer Mix 10 µM, and 10.5 µL of nuclease-free water. The expression level of mRNA was quantified by qRT-PCR using the QuantStudio 12K Flex Real-Time PCR System (ThermoFisher). The housekeeping gene ABL1 was used for data normalization, and differential gene expression was determined using the comparative ΔΔCt method, in which gene expression levels are normalized in relation to a household gene, and then are compared between cell lines and different conditions (Figure 3A). For the basal expression levels of SMPD genes in MM cell lines (Figure 2A), the -dCt value is portrayed. 

### 4.9. Western Blotting 

HMCL was seeded in a 6-well plate and cultured during 24 h with medium without serum, melphalan, or bortezomib, with or without amitriptyline at the same concentrations used for the viability assay. Cells were lysed in lysis buffer containing 50 mM Tris, 150 mM NaCl, 1% Nonidet P40, and 0.25% sodium deoxycholate. The following protease and phosphatase inhibitors were added: 4 mM Na3VO4, 1 mM Na4P2O7, 2 µg/mL aprotinin, 50 µg/mL leupeptin, 500 µg/mL trypsin inhibitor, 10 µM benzamidine, 2.5 mM pnp benzoate (all from Sigma-Aldrich), 50 mM NaF, 5 mM ethylenediaminetetraacetic acid (both from VWR International), 1 mM 4-(2-aminoethyl) benzenesulfonyl fluoride hydrochloride, and 50µg/mL pepstatin A (both from ICN). 

Western blot analysis on these cell lysates was performed as previously described [13]. Chemiluminescence was visualized and analyzed using a Li-Cor Odyssey Fc. Quantification was performed using Image Studio Lite version 5.2 software.

Antibodies used for analysis were: ASM (#3687), β-ACTIN (#4967), PARP (#9542), Caspase 3 (#9665), all from Cell Signaling Technology. For exosome characterization, we used CD63 (H-193, sc-15363), and CD81 (B-11, sc-166029) from Santa Cruz Biotechnology (Heidelberg, Germany).

### 4.10. Total SMase Measurement in Samples

Total SMase activity was measured by an Amplex Red Sphingomyelinase Assay Kit (A12220, Thermo Fisher, Waltham, MA, USA) using the continuous assay, according to the manufacturer’s instructions. Briefly, diluted samples and controls were added in a 96-well plate, after which the working solution containing Amplex Red reagent was added to these samples. After three hours of incubation at 37 °C, fluorescence was measured using a Glomax fluorometer (Promega).

SMase activity was determined in supernatants and lysates of HMCL and on plasma samples according to the manufacturer’s conditions. 

### 4.11. Sphingomyelin Assay

HMCL was seeded in a 6-well plate and cultured during 24 h with medium without serum, melphalan or bortezomib, with or without amitriptyline at the same concentrations used for the viability assay. The supernatant was collected and analyzed for sphingomyelin content using a fluorometric Sphingomyelin Assay Kit (ab138877, Abcam, Cambridge, UK). Fluorescence was measured after 2 h of incubation at room temperature using a Glomax fluorometer (Promega), according to the manufacturer’s instructions. 

### 4.12. Statistical Analysis

Results were analyzed with GraphPad Prism 5.0 software (GraphPad Software Inc, La Jolla, CA, USA). All data represent the mean ± standard deviation (SD), and results were analyzed using the Mann–Whitney U test and one-way ANOVA (combination indexes, CI). *p* < 0.05 (*), *p* < 0.01 (**), and *p* < 0.001 (***) were considered statistically significant.

## 5. Conclusions

In conclusion, in this paper, we analyzed for the first time the differences in the lipid profiles of myeloma patients and could correspond the imbalance in ceramide and sphingomyelin to changes in ASM levels in MM cells. Furthermore, we saw an upregulation in ASM expression in MM cells and their exosomes upon exposure to anti-myeloma drugs. Exosomes high in ASM content were able to transfer the resistant nature of their cells of origin unto other, initially sensitive MM cells. Importantly, amitriptyline and GW4869, as inhibitors of SMase, are able to increase sensitivity to standard-of-care anti-myeloma drugs, providing a rational to use SMase targeting drugs in combination strategies for MM patients (graphical abstract).

## Supplementary Materials

The following are available online at https://www.mdpi.com/2072-6694/11/12/1823/s1, Figure S1: Alterations in the MM lipidome, Figure S2: Exosomes confer resistance to MM cells, Figure S3: Effects of amitriptyline and GW4869 on MM cells, Figure S4: Whole western blots with molecular weights. Table S1: Patient and disease characteristics are shown for all Multiple Myeloma patients (n = 38) who were included in the lipidomic analysis.
