# Supplementary material for: The Transfer of Sphingomyelinase Contributes to Drug Resistance in Multiple Myeloma
Source: Cancers (Basel). 2019 Nov 20;11(12):1823. doi: 10.3390/cancers11121823 (PMC6966559; doi:10.3390/cancers11121823)
Supplement: Supplementary file 1 [file cancers-11-01823-s001.pdf]

Supplementary Materials:

The Transfer of Sphingomyelinase Contributes to Drug Resistance in Multiple Myeloma

Sylvia Faict, Inge Oudaert, Ludovic D'Auria, Jonas Dehairs, Ken Maes, Philip Vlummens, Kim De Veirman, Elke De Bruyne, Karel Fostier, Isabelle Vande Broek, Rik Schots, Karin Vanderkerken, Johannes V. Swinnen and Eline Menu

Table 1. Patient and disease characteristics are shown for all Multiple Myeloma patients (n = 38) who were included in the lipidomic analysis.

**Characteristic**	**Categories**	**Number (Percent)**
Age in years	Mean	70,6 y
	Median	71 y
	Range	40–84 y
Prior treatment history	Newly diagnosed	19 (50)
	Relapsed/Refractory	19 (50)
Gender	Male	22 (58)
	Female	16 (42)
ISS-stage	1	13 (35)
	2	18 (47)
	3	7 (18)
Monoclonal protein	IgG	22 (58)
	IgM	0 (0)
	IgA	7 (18)
	unknown	9 (24)
Monoclonal protein (light chain)	κ	17 (45)
	λ	12 (31)
	unknown	9 (24)

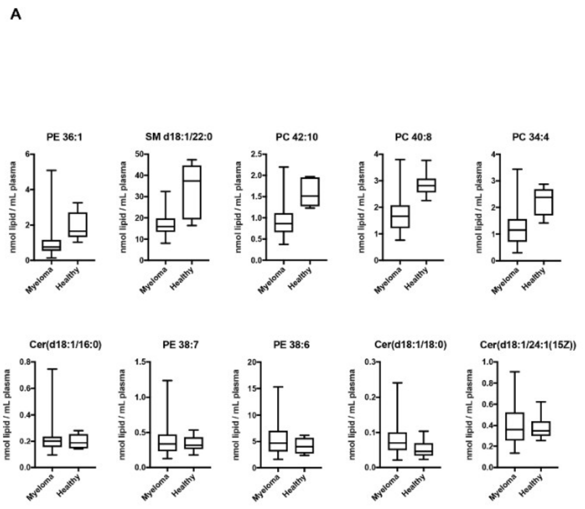

	% of class	nmol /mL plasma		% of class	nmol /mL plasma
SM d18:1/16:0	21%	55.4	Cer(d18:1/16:0)	12,0%	0,22
SM d18:1/24:1	18%	47.8	Cer(d18:1/24:1)	22,1%	0,40
SM d18:1/20:0	9%	24.2	Cer(d18:1/20:0)	3,3%	0,06
SM d18:1/22:1	9%	24.1	Cer(d18:1/22:1)	1,1%	0,02
SM d18:2/24:1	9%	23.2	-	-	-
SM d18:1/22:0	7%	19.1	Cer(d18:1/22:0)	15,5%	0,28
SM d18:1/24:0	6%	15.0	Cer(d18:1/24:0)	38,5%	0,69
SM d18:1/18:0	4%	11.3	Cer(d18:1/18:0)	4,2%	0,08
SM d18:1/16:1	3%	7.6	-	-	-
SM d18:2/22:1	2%	6.3	-	-	-
SM d18:3/24:1	2%	6.1	-	-	-
SM d18:1/18:1	2%	5.3	-	-	-
SM d18:3/22:1	2%	5.2	-	-	-
SM d18:4/24:1	2%	4.2	-	-	-
SM d18:1/20:1	1%	4.0	-	-	-
SM d18:0/22:0	1%	3.2	-	-	-
SM d18:0/16:0	1%	2.9	-	-	-
SM d18:0/18:0	1%	1.6	-	-	-
SM d18:0/24:0	1%	1.5	-	-	-
SM d18:1/26:1	0%	0.6	Cer(d18:1/26:1)	2,6%	0,05
SM d18:1/26:0	0%	0.3	Cer(d18:1/26:0)	0,7%	0,01

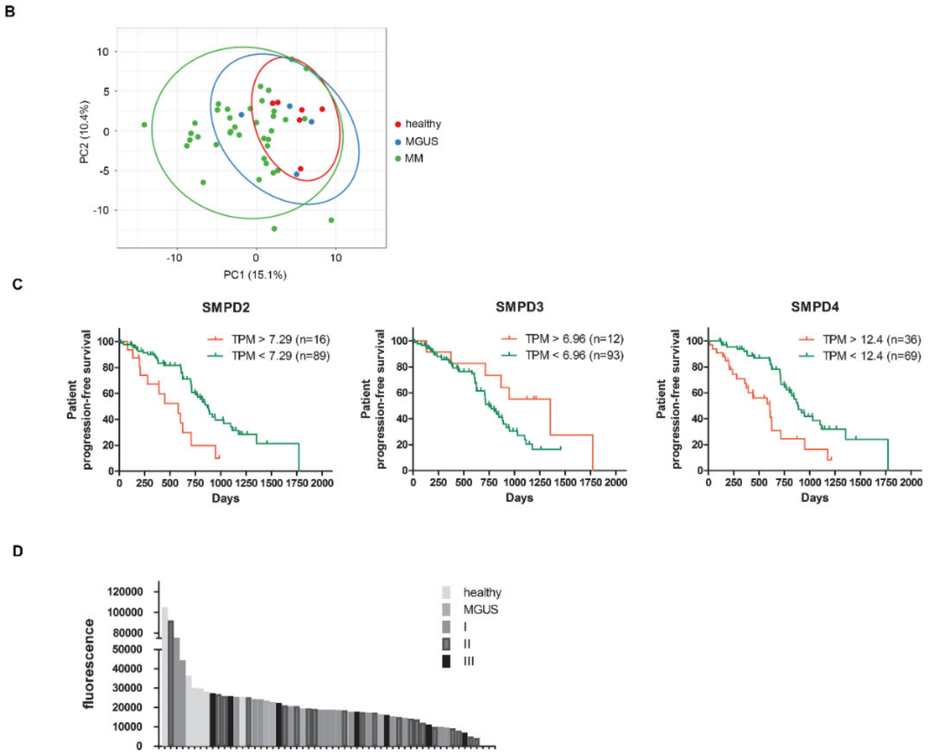

Figure 1. Alterations in the MM lipidome. (**A**) Box plots with the concentrations of the discussed lipid species, ranging from high to low abundance and table with the concentrations and frequency of each sphingomyelin and corresponding ceramide species. (**B**) Principal component analysis for lipidome of plasma from healthy (n = 6), MGUS (n = 4), and MM (n = 42) reveal three groups. The lipidome of MGUS patients seems intermediate to healthy and MM samples. (**C**) Kaplan Meier curve for progression-free survival (PFS) after MaxStat analysis of SMPD2-4 expression in MM patients (MMSET subgroup). (**D**) Waterfall plot of total SMase of individual patients, arranged from high to low and divided according to ISS staging.

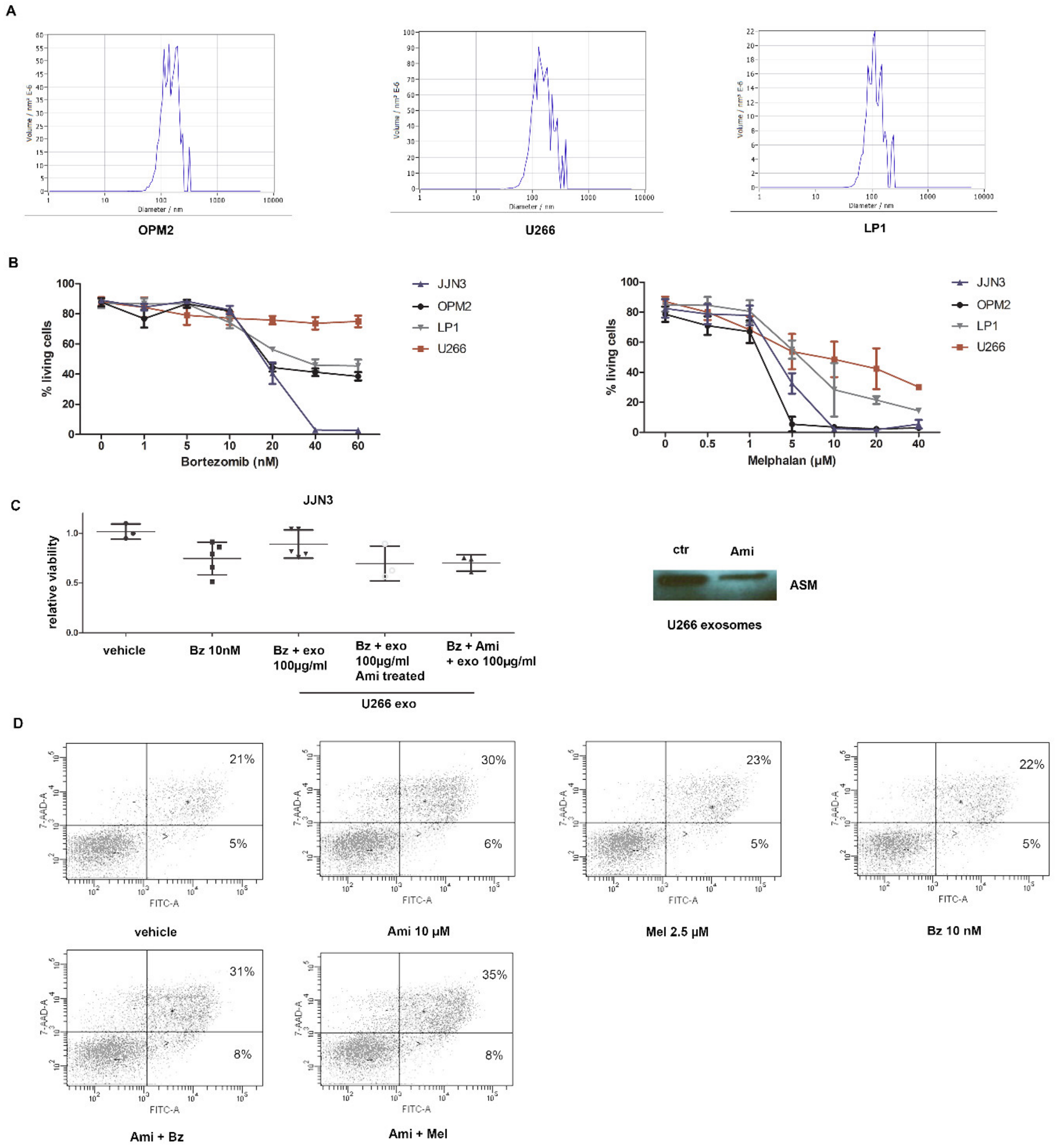

Figure 2. Exosomes confer resistance to MM cells. (**A**) Nanoparticle Tracking Analysis by Zetaview Analysis of exosomes from U266, LP1 and OPM2 cells, confirming the expected size of exosomes. (**B**) Comparison of the sensitivity of different MM cell lines (JJN3, OPM2, LP1 and U266) to melphalan and bortezomib. After 24h of culture, apoptotic cell levels were measured by flow cytometry with staining for 7-AAD and annexin V-FITC. (**C**) Left panel. JJN3 cells were treated with 10 nM of bortezomib with or without previously isolated U266 exosomes. In the last two conditions, U266 cells were either treated with 20 µM amitriptyline prior to exosome isolation or JJN3 cells were treated with 10 µM amitriptyline together with bortezomib. After 24h of culture, cell viability was measured by CellTiterGlo. Right panel. Western Blot demonstrating a reduction of ASM in U266 exosomes treated with amitriptyline. (**D**) Representative dotplots of 7-AAD and annexin V-FITC stainings of JJN3 cells treated with melphalan, bortezomib and/or amitriptyline. ASM = acid sphingomyelinase, Ami = amitriptyline, Mel = melphalan, Bz = bortezomib. The averages of n > 3 are shown.

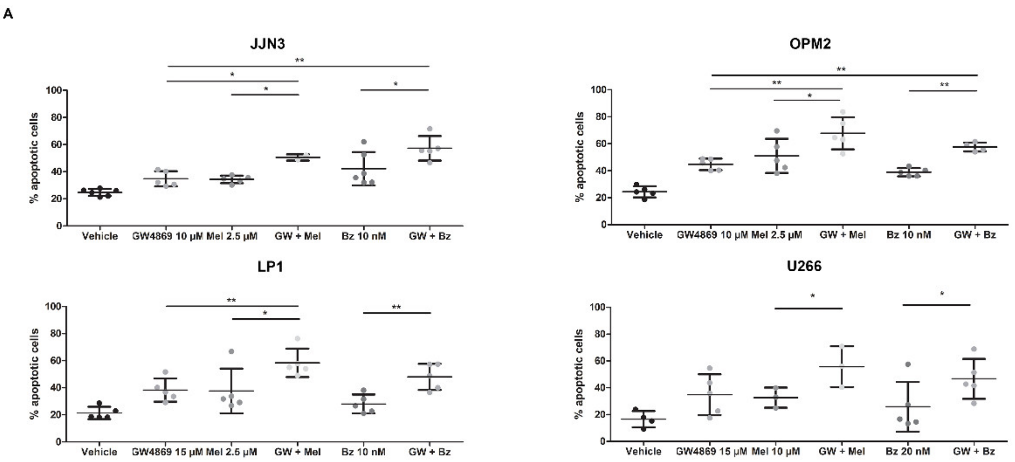

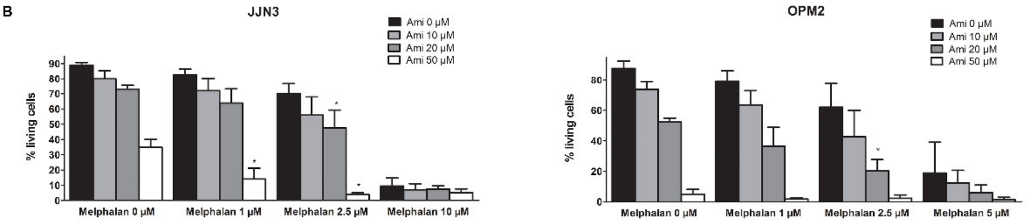

Ami (µM)	Melphalan (µM)	Effect	CI
10	1	0,28	1,19
10	2,5	0,44	1,31
10	10	0,93	0,77
20	1	0,36	1,38
20	2,5	0,53	1,33
20	10	0,93	0,84
50	1	0,86	0,47
50	2,5	0,96	0,24
50	10	0,94	0,76

Ami (µM)	Melphalan (µM)	Effect	CI
10	1	0,42	1,31
10	2,5	0,65	1,50
10	5	0,89	1,29
20	1	0,71	1,26
20	2,5	0,81	1,40
20	5	0,94	1,21
50	1	0,98	0,75
50	2,5	0,97	1,07
50	5	0,98	1,15

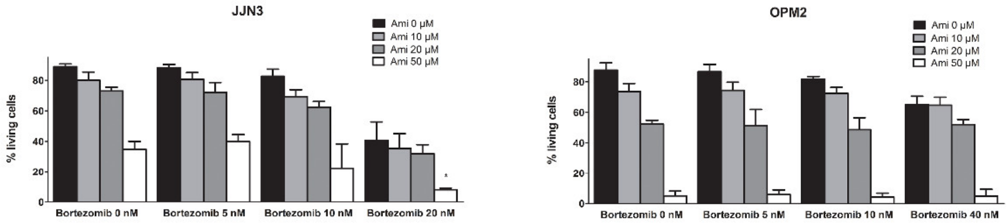

Ami (µM)	Bortezomib (nM)	Effect	CI
10	5	0,19	1,52
10	10	0,31	1,42
10	20	0,65	0,95
20	5	0,28	1,70
20	10	0,38	1,60
20	20	0,68	1,02
50	5	0,60	1,28
50	10	0,78	0,82
50	20	0,92	0,49

Ami (µM)	Bortezomib (nM)	Effect	CI
10	5	0,29	1,08
10	10	0,30	1,23
10	40	0,36	1,67
20	5	0,55	1,12
20	10	0,55	1,21
20	40	0,49	1,57
50	5	0,94	1,10
50	10	0,96	0,94
50	40	0,94	1,16

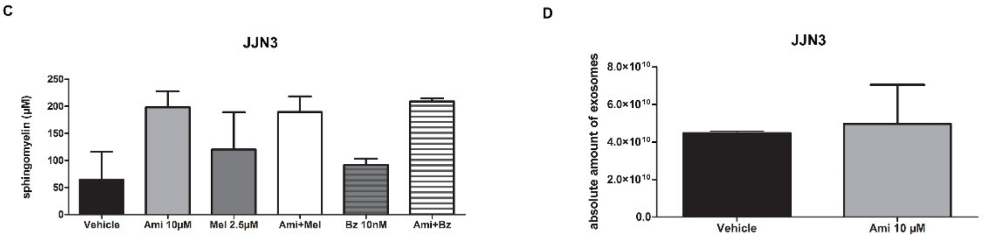

Figure 3. Effects of amitriptyline and GW4869 on MM cells. (**A**) GW4869, a neutral SMase inhibitor, is also able to increase drug efficacy of melphalan and bortezomib in MM cells. JJN3, OPM2, LP1 and U266 were treated with melphalan, bortezomib and/or GW4869. After 24h of culture, apoptotic cell levels were measured by flow cytometry with staining for 7-AAD and annexin V-FITC. (**B**) JJN3 and OPM2 cells were treated with varying concentrations of amitriptyline, bortezomib and melphalan for

24h. Apoptotic cells were measured by flow cytometry with staining for 7-AAD and annexin V-FITC. Combination indexes were calculated for the combination therapy of melphalan/amitriptyline and bortezomib/amitriptyline. CI < 0,8 was considered a synergistic effect while CI < 1,2 was considered an additive effect. * indicates significance compared to both single agents alone. (**C**) Analysis of the amount of sphingomyelin in the supernatant of JJN3 cells treated with melphalan/bortezomib with or without amitriptyline. As expected, sphingomyelin is accumulated when cells are treated with amitriptyline because of an inhibition of ASM. (D) A. Nanoparticle tracking analysis shows the amount of exosomes secreted after 24h treatment with amitriptyline 10 µM in JJN3 cells. $p < 0.05$ (*), and $p < 0.01$ (**) were considered statistically significant. The averages of $n > 3$ are shown.

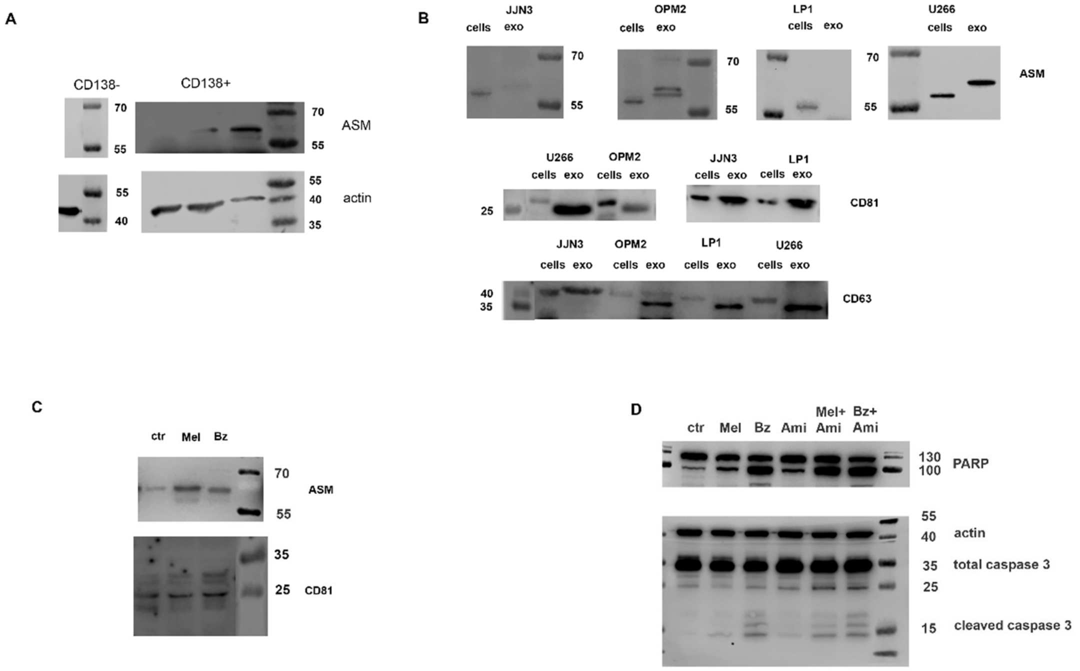

Figure S4. Whole Western Blots with molecular weights. **(A)** Representative western blot for figure 1D. (**B**) Representative western blot for figure 2C. (**C**) Representative western blot for figure 3C. (**D**) Representative western blot for figure 4B.
